# Simultaneous effects on parvalbumin-positive interneuron and dopaminergic system development in a transgenic rat model for sporadic schizophrenia

**DOI:** 10.1038/srep34946

**Published:** 2016-10-10

**Authors:** Hannah Hamburg, Svenja V. Trossbach, Verian Bader, Caroline Chwiesko, Anja Kipar, Magdalena Sauvage, William R. Crum, Anthony C. Vernon, Hans J. Bidmon, Carsten Korth

**Affiliations:** 1 Institute of Neuropathology, Heinrich Heine University Düsseldorf, Germany; 2 Cécile and Oskar Vogt Institute of Brain Research, Heinrich Heine University Düsseldorf, Germany; 3 Institute of Veterinary Pathology, Vetsuisse Faculty, University of Zurich, Switzerland; 4 Functional Architecture of Memory Unit, Mercator Research Group, Faculty of Medicine, Ruhr University Bochum, Bochum, Germany; 5 King’s College London, Institute of Psychiatry, Psychology and Neuroscience, Department of Neuroimaging, London, United Kingdom; 6 King’s College London, Institute of Psychiatry, Psychology and Neuroscience, Department of Basic and Clinical Neuroscience, London, United Kingdom

## Abstract

To date, unequivocal neuroanatomical features have been demonstrated neither for sporadic nor for familial schizophrenia. Here, we investigated the neuroanatomical changes in a transgenic rat model for a subset of sporadic chronic mental illness (CMI), which modestly overexpresses human full-length, non-mutant Disrupted-in-Schizophrenia 1 (DISC1), and for which aberrant dopamine homeostasis consistent with some schizophrenia phenotypes has previously been reported. Neuroanatomical analysis revealed a reduced density of dopaminergic neurons in the substantia nigra and reduced dopaminergic fibres in the striatum. Parvalbumin-positive interneuron occurrence in the somatosensory cortex was shifted from layers II/III to V/VI, and the number of calbindin-positive interneurons was slightly decreased. Reduced corpus callosum thickness confirmed trend-level observations from *in vivo* MRI and voxel-wise tensor based morphometry. These neuroanatomical changes help explain functional phenotypes of this animal model, some of which resemble changes observed in human schizophrenia *post mortem* brain tissues. Our findings also demonstrate how a single molecular factor, DISC1 overexpression or misassembly, can account for a variety of seemingly unrelated morphological phenotypes and thus provides a possible unifying explanation for similar findings observed in sporadic schizophrenia patients. Our anatomical investigation of a defined model for sporadic mental illness enables a clearer definition of neuroanatomical changes associated with subsets of human sporadic schizophrenia.

To date, neuropathological features correlating to the exclusive clinical diagnosis “schizophrenia” are controversial, likely due to 1) the subtlety of changes, 2) the heterogeneity of underlying biological causes, and 3) the high inter-subject variability of brain microanatomy in human individuals. Efforts to characterize morphological changes have been made since the clinical description of the condition utilizing both *in vivo* imaging as well as *post mortem* approaches.

Even though the clinical success of dopamine antagonists in treating the acute psychotic phase of schizophrenia in a majority of patients has led to the biological “dopamine hypothesis” of schizophrenia (reviewed in ref. 1) and thus helped to define a common ground for this clinical syndrome, neuropathological investigations have failed to clearly define phenotype - clinical disease correlations^2^,^3^,^4^. This is in contrast to, for example, the classification of neurodegenerative diseases, where, apart from distinctive neuronal cell death, each category can be defined by the accumulation or disturbed proteostasis of specific proteins. Strikingly, the very same proteins that accumulate in the majority of sporadic cases of neurodegenerative diseases are the same as those which are mutated in the familial cases that usually make less than 5% of all cases^5^. Notably, however, any neuropathological changes in schizophrenia are likely to be more subtle than those of neurodegenerative diseases and do not comprise significant cell death^6^.

One consistent but unspecific phenotype of schizophrenia patients are enlarged ventricles. For example, a recent prospective large-scale meta-analyses of MRI data from more than 2000 cases of schizophrenia and healthy controls each via the ENIGMA consortium confirmed an approximate ~19% increase in ventricular volume of schizophrenia patients with medium effect size (Cohen’s d = 0.37)^7^.

Furthermore, a decrease in cortical volume and disproportionate volume loss of temporal structures have been described (reviewed in ref. 4). Regarding white matter alterations, the corpus callosum is one of the structures which most commonly shows abnormalities^8^,^9^ with several studies reporting a reduction of its volume^10^,^11^,^12^.

Early MRI analyses assessing striatal size because of its major dopaminergic input and relevance in cognitive, sensory and motor processing yielded mixed results, with the majority of studies reporting an increase in volume (reviewed in ref. 13). Two recent studies have also demonstrated enlarged striatal sizes in drug-naïve patients suffering from either schizophrenia or schizotypal disorders^14^,^15^. Nevertheless, recent analysis of MRI data from the schizophrenia working group of the ENIGMA consortium reported no differences in either caudate or putamen volume in schizophrenia patients relative to healthy controls, although increased putamen volumes were associated with duration of illness and age^16^. This corroborates the findings reported in a large-scale meta-analysis of MRI data (18,000 subjects) by Haijma and colleagues^17^ who observed lower caudate volumes only in medication-naïve individuals with schizophrenia compared with controls. Importantly, cumulative exposure to antipsychotic drugs has been linked to either increase or normalization of caudate and putamen volumes, as well as cortical thinning in clinical MRI studies of schizophrenia patients^17^ and pre-clinical MRI studies of antipsychotic drug effects on brain volume^18^,^19^, making it difficult to always distinguish definitively between drug effects and disease effects in medicated schizophrenia patients.

Alterations in the number and positioning of inhibitory GABAergic neurons have repeatedly been reported to be a phenotype of schizophrenia (reviewed in refs 20 and 21). The most consistent change is a reduced number of interneurons, especially parvalbumin (PV) expressing cells which provide the main inhibitory control for pyramidal output neurons (reviewed in ref. 2).

The conundrum is that even though it is generally recognized that schizophrenia comprises heterogeneous biological causes converging on a final common, stereotypical behavioural pathway, all cases of schizophrenia are still treated as one entity in clinical as well as research practice. This clearly provides the risk of diluting potentially different anatomical/neuropathological phenotypes of subsets in investigations under the common umbrella of clinically defined “schizophrenia”. In addition, the considerable background of microanatomical inter-subject variability between humans renders investigations of subtle microanatomical changes difficult^22^,^23^. It is also not clear how the different reported changes could eventually hang together. A solution to this dilemma may be, rather than searching for a common denominator to *all* clinically defined schizophrenia cases, to instead define subsets of cases with schizophrenia by common biological and neuropathological signatures from the outset.

Within this context it has been revealing to investigate mutant genes, originally identified in families affected with schizophrenia, in genetically modified mouse models. Notably, the mutant *Disrupted-in-schizophrenia 1 (DISC1*) gene has been shown to lead to neurodevelopmental changes consistent with the proposed neurodevelopmental origin of schizophrenia^24^,^25^,^26^. A mutant *DISC1* gene has been identified in two independent families to date^27^,^28^,^29^ and animal models of mutant *DISC1* have consistently revealed, amongst other phenotypes, neuroanatomical changes including altered interneuron distribution, disturbed dopamine signalling and dendritic alterations (reviewed in ref. 30). Furthermore, *in utero* DISC1 knockdown studies have revealed deficits in the migration of pyramidal neurons, a reduced number of PV-interneurons and abnormal maturation of dopaminergic neurons^24^,^31^,^32^ establishing a neurodevelopmental function for DISC1.

DISC1 is known to be involved in the neurodevelopment of cortical progenitor cells via GSK-3β/β-catenin where it acts as a switch from proliferation to migration through a phosphorylation site^33^,^34^. However, it has recently been demonstrated that it is also involved in the tangential migration of cortical interneurons in a regulatory manner^32^. Knockdown of DISC1 leads to a reduction of PV-positive neurons in the cortex by interaction with the actin cytoskeleton during nucleokinesis, leading to impaired tangential migration of interneurons originating from the ganglionic eminence^35^. Reduced numbers or abnormal distribution of PV-positive neurons are a frequent finding in mutant DISC1 mouse models^31^,^36^,^37^.

One characteristic feature of DISC1 mouse models is the enlargement of the lateral ventricles, with some also displaying a concurrent thinning of the cortex^36^,^37^,^38^,^39^,^40^. Further morphological changes include diverse dendritic alterations, such as reduced or enhanced spine density, reduced dendrite length and neurite outgrowth as well as mis-oriented dendrites^31^,^36^,^37^,^41^,^42^,^43^.

To date, partial agenesis of the corpus callosum has been described in one mutant DISC1 mouse model^36^, consistent with the frequent reporting of white matter changes in neuroimaging studies of patients with schizophrenia^9^.

Even though analysis of the effects of mutant genes on neurodevelopment and adult behavioural phenotypes may be revealing, potentially linking the mutant gene to a particular disease-related phenotype, it is not evident how the non-mutant gene could do so in the majority of sporadic cases of schizophrenia, i.e. patients without an obvious genetic defect. One possible way in how non-mutant gene products could nevertheless play a specific role in disease mechanisms would be through posttranslational modifications. In analogy to the fact that proteins subject to proteostatic disturbance in familial cases of neurodegenerative disease are often the same as those in sporadic cases^5^, we previously hypothesized that the DISC1 protein could be insoluble in sporadic cases of chronic mental illness. Indeed, we demonstrated that to be the case in about 10% of *post mortem* brains of patients with chronic psychiatric disease^44^. Modelling the presence of insoluble DISC1 aggregates in a transgenic rat model by modestly overexpressing the full length, non-mutant DISC1 protein, we observed ubiquitous perinuclear DISC1 aggregates that were accentuated in dopamine-rich regions such as the dorsal striatum, as well as biochemical and behavioural phenotypes consistent with aberrant dopamine homeostasis^45^. Specifically, this rat model showed decreased total levels of dopamine in the dorsal striatum, the amygdala and the hippocampus, an increased proportion of high-affinity dopamine D2 receptors and translocated dopamine transporter leading to altered dopamine flow dynamics. On the systemic level, an amphetamine supersensitivity was also observed^45^.

Based on this behavioural and neurochemical analysis of the tgDISC1 rat, we hypothesized that morphological irregularities in the central nervous system of tgDISC1 rats would be present. We therefore carried out a detailed *post mortem* microscale examination of brains from tgDISC1 rats, as well as littermate controls (LM). Based on prior observations in this tgDISC1 rat as well as mutant DISC1 models, this *post mortem* analysis initially focused on the dopaminergic system and PV-interneurons as well as the corpus callosum. In parallel, macroscale MRI combined with voxel-wise tensor-based morphometry^19^ were conducted to provide brain-wide mapping of the anatomy of tgDISC1 rats in comparison to LM. Additional *post mortem* analyses were then carried out guided by the additional results arising from the MRI analysis. We found reduced numbers of dopaminergic neurons in the compact part of the substantia nigra (SNC) and sparser dopaminergic fibre density in the striatum. The distribution of PV-positive neurons was shifted to the deeper cortex layers and the corpus callosum showed a volumetric decrease in tgDISC1 rats, when compared to LM.

## Results

### Modest overexpression of full-length human DISC1 reduces TH-positive fibre density in the striatum and enlarges the striosomal compartment in tgDISC1 rats

**Figure 1:**
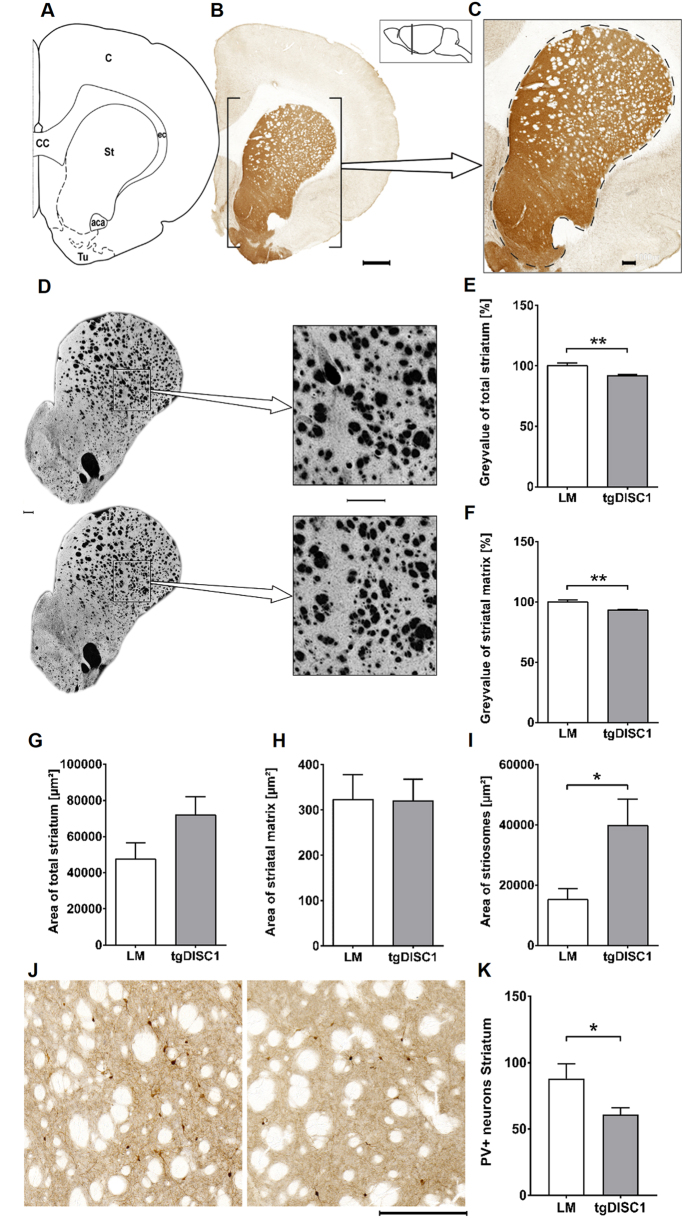
Dopaminergic fibre density and striosomal surface in the striatum. (**A**) Schematic figure highlighting the relevant anatomical structures of (**B**) a representative section, bar 1000 μm. (**C**) A close-up is displayed illustrating the striatum with a dashed line. Grey value and area measurements were obtained within this labelled ROI. The position with respect to bregma is indicated in the schematic (lateral) brain illustration, bar 250 μm. (**D**) Exemplary TH-immunostained striatal sections of each group with the upper specimen originating from a tgDISC1 rat and the lower specimen from a littermate control. Colours were inverted to better illustrate the fibre density, and close-ups are displayed on the right. Note the subtly sparser matrix and increase of striosomal surface in the tgDISC1 rat. Bars 250 μm. (**E**) Grey value of the entire striatum. TgDISC1 rats display lower grey intensity in the entire striatum (*p* = 0.005). (**F**) Grey value of the striatal matrix without striosomes. TgDISC1 rats have a lower grey intensity in the striatal matrix (*p* = 0.004). (**G**) Area of the entire striatum including matrix and striosomal compartment. No difference in the surface area is detected between the two groups. (**H**) Area of the striatal matrix. No difference in the surface area of the striatal matrix is detected between the two groups. (**I**) Area of striosomes. TgDISC1 rats have an increased striosomal area (*p* = 0.038). (**J**) Magnification of the dorsal striatum of a LM on the left and a tgDISC1 rat on the right, note the sparser PV-positive interneurons in the tgDISC1 rat, bar 250 μm. (**K**) PV-positive interneurons in the dorsal striatum. PV-interneuron number is decreased in tgDISC1 rats (p = 0.047). Surface area is displayed in μm^2^ ± s.e.m. and normalized reciprocals of the grey values are displayed in percent ± s.e.m. Abbreviations: anterior part of the anterior commissure (aca), cortex (C), corpus callosum (cc), external capsule (ec), striatum (St), olfactory tubercle (Tu).

Since we had previously observed aberrant dopamine homeostasis in the tgDISC1 rat, accentuated in the striatum^45^, the histomorphology of the striatum of the tgDISC1 rat was analyzed by comparing striatal size and tyrosine hydroxylase (TH)-positive fibre density of tgDISC1 rats vs. non-transgenic LM (Fig. 1A–C). Grey value for a precise estimation of TH-positive fibres as well as area measurements were performed on TH-immunostained brain sections. Analysis was carried out on the striatum as a whole and also on both striatal compartments: the matrix and the striosomes.

TgDISC1 rats showed a decrease in grey value intensity following TH immunostaining when compared to LM in measurements of the entire striatum (*p* = 0.005, Cohen’s d = 1.66) as well as in the matrix exclusively without the striosomes (*p* = 0.004, Cohen’s d = 1.73), indicating sparser TH-positive fibres at this location (Fig. 1D–F). There was no significant difference in the total area of the striatum, but there was a trend towards an increase in transgenic animals (Fig. 1G). Looking at the individual compartments, the size of the striatal matrix did not differ between the two groups, whereas a striosomal expansion was present in tgDISC1 rats (*p* = 0.038, Cohen’s d = 1.15, Fig. 1H,I). Therefore, our morphological analysis indicates that tgDISC1 rats display lower TH-positive fibre density, resulting from both fewer absolute numbers of TH-positive fibres and enlargement of the striosomal compartment.

### Decreased numbers of TH-positive neurons in the SNC but not in the VTA of tgDISC1 rats

**Figure 2:**
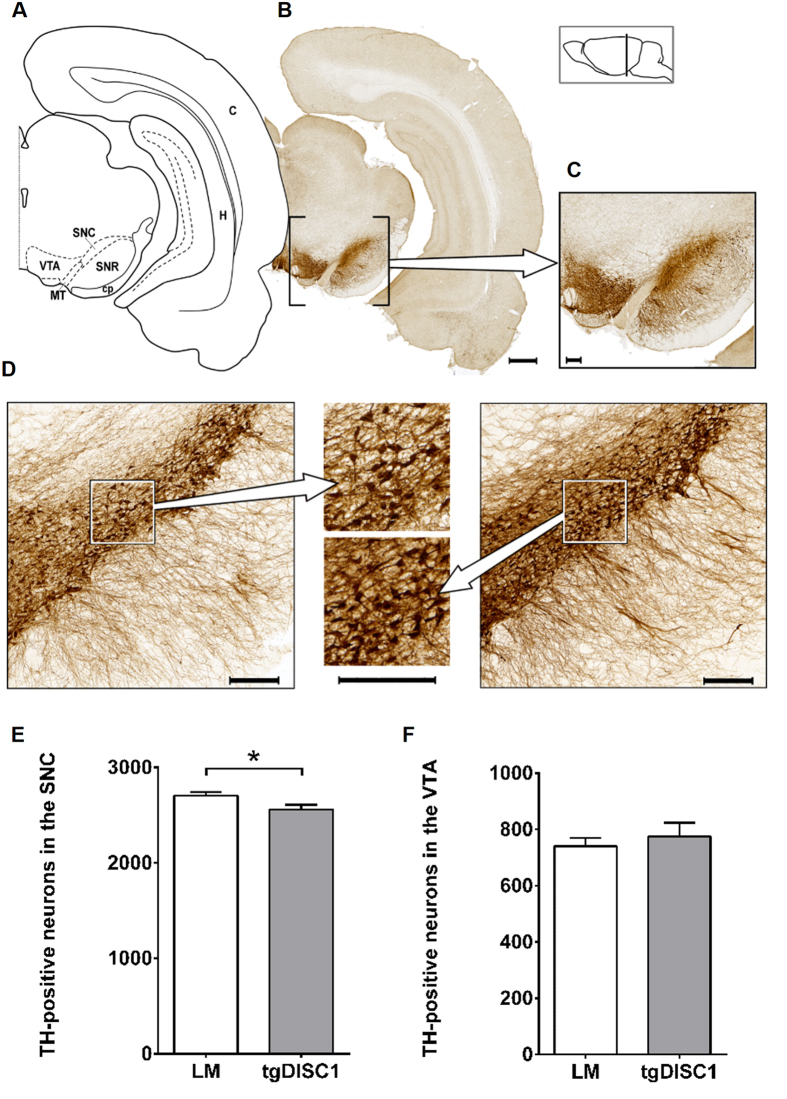
Tyrosine Hydroxylase-positive neurons in the substantia nigra pars compacta and the ventral tegmental area. (**A**) Schematic figure highlighting the relevant anatomical structures of a (**B**) TH-immunostained exemplary section, bar 1000 μm. (**C**) A close-up of these nuclei is displayed, bar 250 μm. The position with respect to bregma is indicated in the lateral schematic illustration. (**D**) Magnification of the SN of a tgDISC1 rat on the left and a LM on the right with magnifications displayed in the centre. Note the sparser TH-positive neurons in the SN of the tgDISC1 rat. Bars are 250 μm. (**E**) TH-positive neurons in the SNC. TgDISC1 rats have a decreased number of TH-positive neurons in the SNC (*p* = 0.037) compared to littermate controls. (**F**) TH-positive neurons in the VTA. No difference in quantity of TH-positive neurons is observed in the VTA (*p* = 0.557). Neuronal cell counts are displayed in number of cells ± s.e.m. Abbreviations: cortex (C), cerebral peduncule (cp), hippocampus (H), medial terminal nucleus of the accessory optical tract (MT), substantia nigra, pars compacta (SNC), substantia nigra, pars reticulate (SNR), ventral tegmental area (VTA).

Dopaminergic fibres of the nigrostriatal, mesolimbic and mesocortical pathway arise from neurons in the SNC and the ventral tegmental area (VTA), innervating the dorsal striatum as well as the nucleus accumbens and cortex, respectively^46^. To assess potential alterations in dopaminergic neuron density in these regions, we performed a neuronal cell count on TH-immunostained brain sections from both tgDISC1 rats and LM. Transgenic animals showed a decrease in TH-positive neurons in the SNC (*p* = 0.037, Cohen’s d = 1.15) but not in the VTA (*p* = 0.557, Fig. 2). Of note, DISC1 is expressed in the SN as well as the VTA (Supplementary Figure 1).

### Dopamine fibre density in the amygdala is not affected by DISC1 overexpression

**Figure 3:**
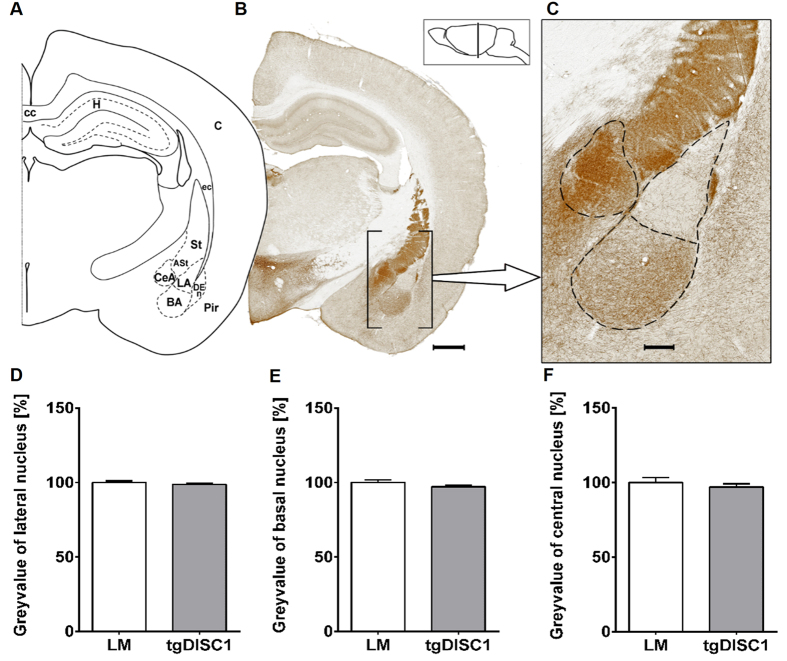
Dopaminergic fibre density in the amygdala. (**A**) Schematic figure highlighting the relevant anatomical structures of (**B**) a TH-immunostained exemplary section, bar 1000 μm. (**C**) A close-up is displayed illustrating the amygdalar nuclei with a dashed line. Grey value and area measurements were carried out in the lateral, the basal and the central nucleus. The position with respect to bregma is indicated in the lateral schematic illustration, bar 250 μm. (**D**–**F**) Grey value of the lateral, basal and central nucleus, respectively. No differences in grey value of the nuclei of the amygdala are detected between the two groups. The normalized reciprocals of the grey value are displayed in percent ± s.e.m. Abbreviations: amygdalostriatal transition area (ASt), basal amygdalar nucleus (BA), cortex (C), corpus callosum (cc), central amygdalar nucleus (CeA), external capsule (ec), dorsal endopiriform nucleus (DEn), Hippocampus (H), lateral amygdalar nucleus (LA), piriform cortex (Pir), striatum (S).

The amygdala plays a major role in processing affective and especially threatening stimuli as well as emotional memory consolidation. It receives its dopaminergic input from the VTA. To determine whether changes in dopamine fibre density, such as those observed in the striatum, were also present in the amygdala, we examined the size and TH-positive fibre density within the amygdalar complex (Fig. 3A–C). The amygdala was divided into lateral, basal, and central nuclei.

Grey value measurement showed no differences between tgDISC1 rats and LM (Fig. 3D–F). However, tgDISC1 rats displayed a slight but non-significant trend towards an increased size of the basal nucleus and a trend towards a slight shrinkage of the central nucleus (Supplementary Figure 2).

### Altered PV-interneuron distribution in the cortex, but no differences in cortical layer thickness in the tgDISC1 rat

**Figure 4:**
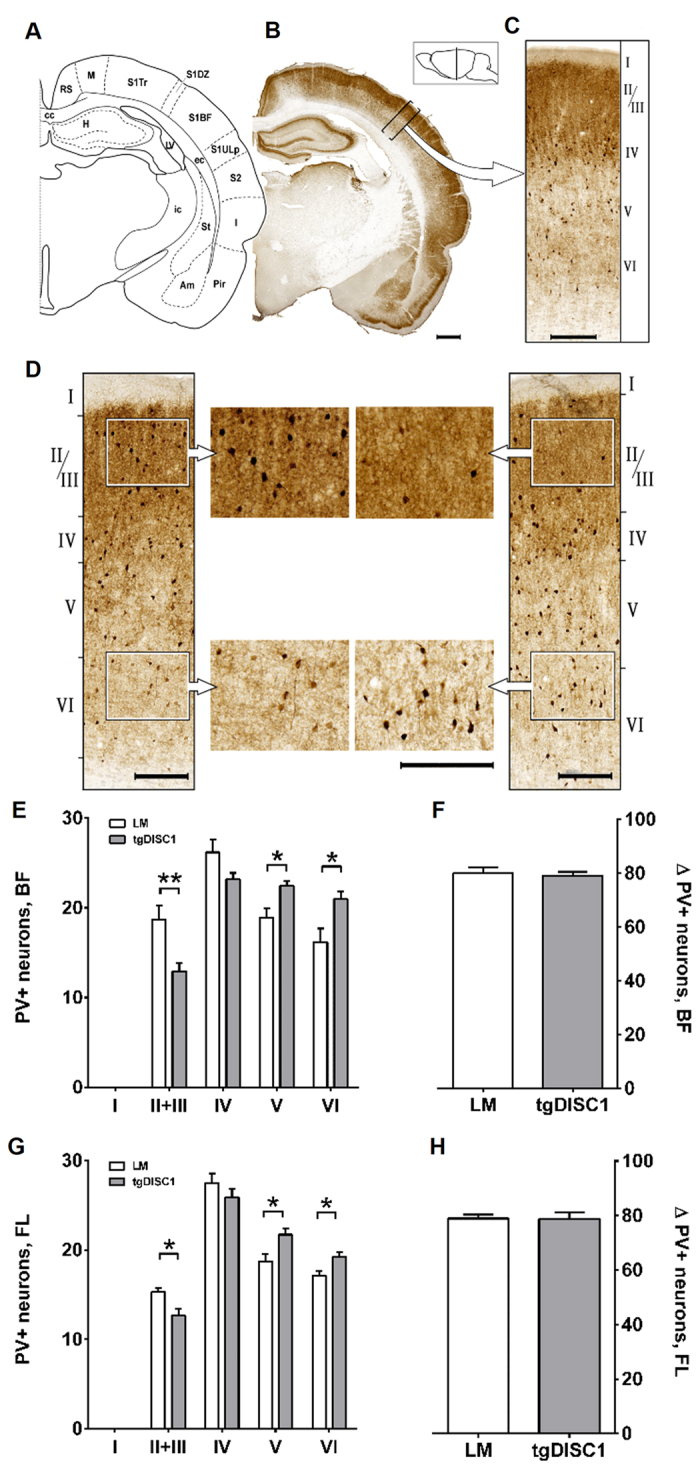
Distribution of Parvalbumin (PV)-positive interneurons in the SSC and cortex layer thickness. (**A**) Schematic figure highlighting the relevant anatomical structure of (**B**) a PV-immunostained exemplary section, bar 1000 μm. (**C**) A close-up of a ROI of the barrel field is illustrated, bar 250 μm. ROIs of 500 μm width and variable length covering all cortical layers were placed perpendicularly to the white matter and interneurons were counted per cortex layer. The position with respect to bregma is indicated in the lateral schematic illustration. (**D**). ROIs from the barrel field of a LM (left panel) and a tgDISC1 rat (right panel) with magnifications in the centre. Note the reduced PV-interneuron number in layers II/III and its increase in layers V/VI, bars 250 μm. (**E**) Cortex thickness including all layers. No difference between the two groups is detected in the thickness of the entire cortex. (**F**) PV-positive neurons per cortex layer in the BF. TgDISC1 rats show a decrease of PV-positive neurons in layer II+III (*p* = 0.005) and an increase in layers V (*p* = 0.011) and VI (*p* = 0.019) compared to littermate controls. (**G**) Total cell count of PV-positive neurons in the BF. No difference in total number of PV-positive neurons is detected. (**H**) PV-positive neurons per cortex layer in the FL region. In tgDISC1 rats, PV-positive neurons are reduced in layer II + III (*p* = 0.014), but increased in layers V (*p* = 0.022) and VI (*p* = 0.015). (**I**) Total cell count of PV-positive neurons in the FL region. No difference in total number of PV-positive neurons is detected. Layer thickness is displayed in μm ± s.e.m., neuronal cell counts are displayed in number of cells ± s.e.m. Abbreviations: amygdala (Am), corpus callosum (cc), external capsule (ec), hippocampus (H), insular cortex (I), lateral ventricle (LV), motor cortex (M), piriform cortex (Pir), retrosplenial cortex (RS), barrel field of the primary somatosensory cortex (S1BF), dysgranular zone of the primary somatosensory cortex (S1DZ), trunk region of the primary somatosensory cortex (S1Tr), upper lip region of the primary somatosensory cortex (S1ULp), secondary somatosensory cortex (S2).

To determine whether DISC1 overexpression causes alterations in quantity or distribution of immunolabelled interneurons, we examined cortical layer thickness as well as Glutamate Decarboxylase 67 (GAD67), PV- and calbindin (CB)-positive interneuron numbers and distribution in tgDISC1 rats compared to LM (Fig. 4A–C). Analysis was performed in the primary somatosensory cortex (S1), specifically in both the forelimb region (S1FL) and the barrel field (S1BF). Of note, changes in interneuron frequency in the somatosensory cortex have been reported in CMI patients^47^,^48^,^49^,^50^. Numbers of interneurons in both groups were compared in each cortical layer as well as the total cell count across all such layers. For the assessment of cortical thickness, adjacent Nissl stained sections were used which is the gold standard method for determining cortical layers in adult animal brains^51^,^52^,^53^. No differences in the cortical thickness were observed between the two groups (Supplementary Figure 3).

In tgDISC1 rats, numbers of PV-positive interneurons were decreased in layers II+III (*p* = 0.005, Cohen’s d = 1.72) and increased in layers V (*p* = 0.011, Cohen’s d = 1.56) and VI (*p* = 0.019, Cohen’s d = 1.69), thus revealing a modified distribution pattern (Fig. 4D–H). This shift of PV-positive interneurons to deeper cortical layers was present in both S1BF as well as in the S1FL region, being more pronounced in the S1BF. However, no changes in the total number of PV-positive interneurons were observed (*p* = 0.963 in the S1FL and *p* = 0.732 in the S1BF, Fig. 4F,H).

CB-positive interneurons had similar distribution patterns in both tgDISC1 rats and LM, however, their total amount was slightly decreased in the S1BF but not in the S1FL of tgDISC1 rats (Supplementary Figure 4). No changes were observed in GAD67-positive interneurons (Supplementary Figure 5), indicating that DISC1 targets only a subpopulation of (PV positive) interneurons.

### Reduced PV-interneurons in the dorsal striatum, but no differences in the hippocampus of tgDISC1 rats

Considering the changes in the dopaminergic projections in the striatum and the aberrant distribution of PV-interneurons, we addressed neuron numbers of PV- and GAD67-interneurons in the striatum. PV-interneurons were significantly reduced in the dorsal striatum (*p* = 0.047, Cohen’s d = 1.4, Fig. 1J,K), unlike GAD67-interneurons that were not affected (Supplementary Figure 5). In the hippocampus, neither group of interneurons was significantly altered, even though there was a trend (p = 0.067) to decreased interneurons in layer in the CA1 subfield of the hippocampus (Supplementary Figure 6).

### Macroscale neuroanatomy of tgDISC1 rats in comparison to LM

**Figure 5:**
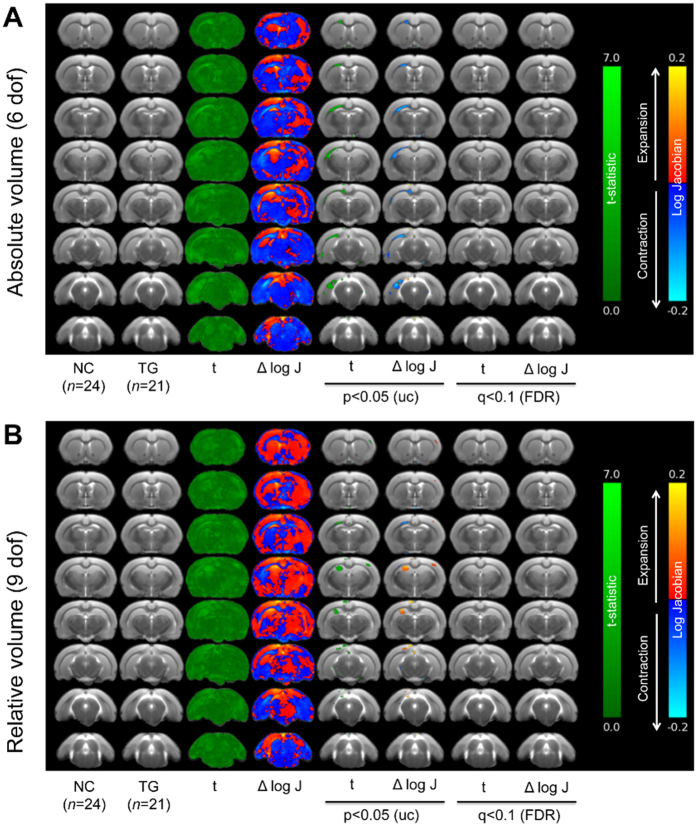
Neuroanatomical differences between LM and tgDISC1 rats assessed through TBM. Maps of voxel-wise local volumetric changes expressed as the delta log scaled jacobian determinant (Δlog J) in male tgDISC1 rats (n = 21) compared to LM (NC; n = 24) at 6 months of age. Images are presented as either (**A**) global volume change (6 dof), or (**B**) absolute changes corrected for global changes (9 dof). Colour scales are for volume difference (cyan/blue to red/yellow) and the raw *t* statistical value at each voxel (dark to light green). Data shown are raw values of Δlog J and their associated *t*-statistic at each voxel and following statistical comparisons at both p < 0.05 uncorrected and following corrections for multiple comparisons (FDR = q < 0.1).

We utilized MR images from live, anaesthized tgDISC1 rats (*n* = 21) and LM (*n* = 24) for automated, voxel-wise, tensor-based morphometry (TBM) analysis to examine neuroanatomical differences on a brain-wide scale. This analysis revealed no significant clusters of volume increase or decrease when comparing tgDISC1 rats and LM after FDR correction (*q* = 0.1), irrespective of controlling for variation in brain volume (Fig. 5A,B). We therefore conducted a second-level analysis at an exploratory threshold of *p* < 0.05 (uncorrected for multiple comparisons). This revealed left-lateralized trend-level decreases in the volume of the corpus callosum and external capsule (Fig. 5A). These remain, although to a reduced extent, when analysis was performed taking into account the variation in whole brain volume (Fig. 5B). The latter analysis also revealed an additional left-lateralized cluster of voxels of increased volume in the hippocampus, particularly, the CA3 sub-field (Fig. 5B). Consistent with the MRI investigations outlined above there were no significant changes in the volume of the cortex when comparing tgDISC1 rats and LM.

### Increased hippocampal volume and decreased corpus callosum volume in tgDISC1 rats

Despite the high power, the TBM analysis findings did not survive statistical correction for multiple comparisons. This may in part be due to the anisotropic 2D nature and low resolution of the MR images acquired, which may affect the precision of our image registration and negatively affect our ability to detect subtle anatomical differences between groups using TBM^54^,^55^. Nevertheless, TBM demonstrated clear trends that might be regarded as “hypothesis generators” to guide further focused postmortem investigations, not based on any *a priori* hypotheses^19^. Therefore, to evaluate the changes in the corpus callosum observed by NMR imaging at a finer scale, we calculated volume estimations using Cavalieri’s principle^56^,^57^ to confirm (or disprove) the trend suggested by the imaging findings. Our calculations confirmed a volumetric reduction of the corpus callosum (*p* = 0.013, Cohen’s d = 1.3). We could also confirm a slight increase in hippocampal volume of tgDISC1 rats (*p* = 0.042, Conhen’s d = 1.3). Due to the fact that TBM results were available after histological preparation of the brains, section sampling of the hippocampus did only partially allow an unequivocal comparison between animals. Therefore, we rather consider this as a supporting trend.

## Discussion

DISC1 is a major vulnerability factor for a wide range of chronic mental illnesses^6^,^58^,^59^,^60^, including schizophrenia, but without a clear segregation to a specific clinical diagnosis. We generated the tgDISC1 rat as a model for a subset of sporadic chronic mental illnesses, in contrast to existing mouse models which variously investigate the Scottish mutation, artificial mutations or partial knockouts of Disc1 (reviewed in ref. 30). The tgDISC1 rat displays protein pathology which is of relevance for sporadic cases of mental illnesses and has a distinct phenotype of aberrant dopamine homeostasis^45^. In this study, we report morphological aberrations in the tgDISC1 rat, both at light microscopical level and macroscale (MRI).

Our analyses revealed a decrease in dopaminergic neurons in the SNC, reduced fibre density in the striatum, and an increase in striosomal surface area. This is consistent with our previous finding of reduced whole dopamine content in the dorsal striatum^45^. Dopaminergic neurons from the SNC send their neurites to the striatum, forming the nigrostriatal pathway^46^. Both this decrease and an increased striosomal surface area seem to be relevant factors leading to a reduced dopaminergic fibre density in the striatum. Imbalances between the striosomal compartment and the matrix have been reported to play a role in various neurological disorders, in which they are believed to be a causal factor of mood dysfunctions^61^. Interference of DISC1 with the development of the dopaminergic system has been demonstrated in various DISC1 mouse models using neurochemistry, biochemistry and behavioural tests (reviewed in ref. 30), however, to the best of our knowledge, in none of these models has a detailed neuroanatomical analysis been performed. Thus we conclude that DISC1 is involved in the development of a mature dopaminergic system and its overexpression and misassembly leads to structural abnormalities in the nigrostriatal pathway in the tgDISC1 rat. At this point, we cannot explain why in the tgDISC1 rat only the SN but not the VTA is affected even though both nuclei stain positive for DISC1 (Figure S1), but the differential effect may hint that additional molecular factors are required for DISC1 to exert the described effect, and that these are differentially present in the SN and the VTA.

Aberrations of interneurons have been reported in several mutant DISC1 mouse models^31^,^36^,^37^, with a reduced number of PV-positive interneurons being the most frequently reported abnormality (reviewed in ref. 30, see also Table 1). Indeed, tgDISC1 rats also show differences when compared to LM. We conducted our analyses in the primary somatosensory cortex as it is a commonly affected site in schizophrenia and recent research emphasised that alterations of this region are specific to schizophrenia^47^. The distribution of PV-interneurons is shifted to the deeper layers in tgDISC1 rats in both the S1BF and the S1FL. Since the thickness of the cortical layers does not change, the abnormal localization pattern is likely the consequence of a migration deficit, as opposed to a layer displacement. The possibility of a mutual relationship existing between altered cortical PV-positive neuronal distribution patterns and alterations in the dopaminergic system cannot be excluded. Furthermore, the PV-interneuron number in the dorsal striatum is reduced.

We did not find significant changes when staining for GAD67 was performed (Supplementary Figure 5) which contrasts to the consistent and positive findings for PV positive interneurons in S1 and the dorsal striatum. For rodents, it has been described that only 40% of GAD67 positive interneurons are also PV positive^62^ meaning that any significant effect in this subpopulation would be diluted out if DISC1 was specific and not a general regulator of interneuron migration, which, as our data show, is not the case. DISC1 overexpression thus specifically affects PV neuron migration. In this context, it is interesting to note that the dominant negative Shen *et al.* mouse^36^ has opposite changes in PV positioning thus setting the dominant negative and overexpressing phenotypes in opposition.

Our observations resemble findings of *post mortem* studies on brains of sporadic schizophrenia patients which consistently reported alterations in GABAergic interneurons. Typically, PV-interneurons are reduced in the middle layers of the cortex, resulting in disturbed microcircuitry^63^,^64^. The reduced PV-interneuron density in the middle layers demonstrated in tgDISC1 rats may potentially have similar effects, resulting in a discrepancy of the most important inhibitory input to pyramidal effector neurons in layers II/III. In contrast, CB-interneurons do not display such prominent changes, with their total number being slightly decreased in the BF. Looking at each individual cortex layer, a very subtle reduction can nevertheless be noted resulting in a discretely declined total cell count. Of note, dopaminergic receptors seem to be selective for PV-interneurons, which could partially explain the more striking abnormalities in that interneuron subgroup^65^.

Our findings here, for technical reasons, focus on changes in the S1 (somatosensory cortex). Even though many investigations in humans report changes in the prefrontal cortex, recent functional imaging data comparing patients suffering from depression or schizophrenia identified S1 as cortical subdivision specifically affected in schizophrenia whereas prefrontal cortex and cuneus were affected in both groups of patients^47^. This highlights that on the neuroanatomical level, the somatosensory cortex has been neglected and our data may encourage more neuroanatomical investigations in that area.

TgDISC1 rats have previously been reported to have enlarged lateral ventricles, based on a blinded manual analysis^45^. These findings are consistent with observations in schizophrenia: ventricular enlargement is a common morphological feature of schizophrenia, while a reduction of the corpus callosum has also been reported in several studies (reviewed in refs 8 and 9). The absence of significance changes in layer thickness makes a significant decrease in layer II/III efferent fibres unlikely and thus sets into focus the possibility of primary changes of white matter. Indeed, in an *in vitro* model, it was suggested that DISC1 has a negative regulatory effect on oligodendrocyte differentiation: DISC1 overexpression impeded their outgrowth, whereas a knockdown promoted it^66^. These *in vitro* findings would be consistent with our *in vivo* findings.

Furthermore, operator-independent, brain-wide TBM analysis of tgDISC1 rats suggests a trend-level reduction in the volume of the corpus callosum and the external capsule, which we could confirm in our subsequent volumetric measurement on a microscopic scale. Indeed, the enlargement of lateral ventricles and the volume of the corpus callosum are mutually dependent variables since an inverse correlation between the volumes of these structures has been recently demonstrated in an MRI study of schizophrenia patients^67^.

In summary, the tgDISC1 rat displays morphological changes in several areas of the brain, including in dopaminergic cells and projections, cortical interneuron positioning and white matter/ventricle volume. While some findings are common among schizophrenia models, morphological changes of dopaminergic cells and PV-interneurons in the tgDISC1 rat seem to be unique among present animal models (Table 1), presumably due to its distinguishing feature of human full-length DISC1 overexpression.

To various extents, similar findings also have been reported in neuroanatomical investigations in *post mortem* brains of patients with schizophrenia. The present results also allow to establish a causal relationship between DISC1 overexpression/misassembly and a variety of seemingly unrelated morphological phenotypes, i.e. decreased dopaminergic cells and fibres, PV-positive interneurons, enlarged ventricles and decreased white matter in the corpus callosum. It is thus conceivable that a single molecular cause can simultaneously be linked to these morphological findings that, in part, have also been reported in patients with schizophrenia. We conclude that the tgDISC1 rat is therefore a face valid animal model for a subset of sporadic schizophrenia, based not only on the described behavioural and physiological phenotype of aberrant dopamine homeostasis but also in terms of its neuroanatomical phenotype.

## Materials and Methods

### Animals

All experiments were conducted in conformity with the Animal Protection Law and were approved by local authorities (LANUV NRW, Recklinghausen, Germany). Male Sprague-Dawley rats were housed under 12 h light/dark conditions with *ad libitum* access to food and water.

### Magnetic resonance imaging

Structural MRI analysis was performed as previously described^45^ on a 7.0 tesla small animal Scanner (Bruker BioSpin, Billerica, MA, USA) with a horizontal bore magnet. We utilized voxel-wise tensor based morphometry (TBM) on coronal scans from tgDISC1 rats (*n* = 21) and LM (*n* = 24) to provide an unbiased, brain-wide overview of neuroanatomical differences between tgDISC1 rats and LM. TBM analysis was performed as previously described^19^,^68^,^69^. Briefly, all brains in the study were first rigidly aligned using an automated intensity-based group-wise registration approach^70^,^71^. A high-dimensional non-rigid registration algorithm was applied to warp each globally aligned scan to the population refs 70,72 and 73. Maps of localized volume difference at each voxel relative to the reference brain were computed from the log of the Jacobian determinant of this non-rigid transformation for each scan. Voxel-wise statistical tests over a brain mask were performed to establish regions of significant volumetric difference between groups. These analyses were performed for both absolute (6 degrees of freedom [dof] registration) and relative (9 dof registration) changes in volume, the latter taking into account population variation in anatomy due to differences in total brain volume^54^. Voxel-wise analyses were corrected for multiple comparisons using the false discovery rate (FDR) correction^74^ at a threshold of q = 0.1 (10%)^55^. Additional exploratory analyses were performed at p < 0.05, uncorrected for multiple comparisons.

### Tissue preparation

Fourteen tgDISC1 rats and fourteen LM aged four to five months were deeply anesthetized with sodium-pentobarbital (70 mg/kg) and transcardially perfused with saline and heparin (400 ml, 10000 IU/l) followed by Zamboni fixative (400 ml, 4% paraformaldehyde and 10% picric acid in PBS, pH 7.4).

Brains were removed and post-fixed in Zamboni fixative for 48 h and incubated in sucrose solution (30% in PBS, pH 7.4) over a period of 48 h for cryoprotection. Subsequently, brains were frozen in isopentane at −40 °C and stored at −80 °C before further processing.

Frozen coronal sections of 50 μm thickness were cut using a microtome cryostat system (Leica SM2000R, Leica Biosystems, Wetzlar, Germany).

For Nissl staining, sections were mounted on tissue slides, air dried, and immersed in 70% ethanol for 12 h. Treatment with 0.1% cresyl violet solution (Sigma-Aldrich, St. Louis, MO, USA) for 20 min was followed by differentiation in 70% ethanol for 10 min. Then, sections were dehydrated in ascending series of ethanol (70% for 10 min, 96% for 20 min and 100% for 30 min), treated with xylene (100%, twice for 10 min each) and covered with coverslips using DPX Mounting medium (Sigma-Aldrich, St. Louis, MO, USA).

Free floating immunohistochemical staining for each marker was carried out in multiwell plates in parallel on tissue sections from all animals.

Sections were initially incubated in blocking solution (10% normal goat serum, Vector laboratories, Burlingame, CA, USA, S-1000 and 0.3% saponin in PBS, pH 7.4) for 2 h at room temperature to reduce non-specific background staining. This was followed by incubation with the primary antibodies diluted in 2% normal goat serum and 0.1% saponin in PBS for 48 h at 4 °C. Anti-tyrosine-hydroxylase-antibody (1:100, mouse-monoclonal, Millipore, Billerica, MA, USA, MAB318), anti-GAD67-antibody (1:500, mouse-monoclonal, Millipore, Billerica, MA, USA, MAB5406), anti-parvalbumin-antibody (1:10.000, mouse-monoclonal, Sigma-Aldrich, St. Louis, MO, USA, P-3171) and anti-calbindin-antibody (1:10.000, mouse-monoclonal, Sigma-Aldrich, C-9848) were used as primary antibodies. Rinsing in PBS (pH 7.4) was carried out three times for 30 min. Biotinylated secondary anti-mouse antibodies (1:500, Dianova, Hamburg, Germany, 115-065-166) diluted in PBS (pH 7.4) were used as secondary antibodies and sections incubated for 24 h at 4 °C, then rinsed in PBS (pH 7.4) three times for 15 min.

Sections were incubated with ABC reagent for 30 min at room temperature (ABC elite kit, 1:300, Vector laboratories, Burlingame, CA, USA, PK-6100) and washed out for 15 min using PBS (pH 7.4), followed by incubation with trisamine buffer twice for 15 min (pH 8.1, 0.05 M, Trizma^®^ base, Sigma-Aldrich, T1503).

Sections were incubated with diaminobenzidine (DAB) (0.05% DAB in trisamine buffer, pH 7.6) for 10 min and hydrogen peroxide (1% H_2_O_2_, resulting in a final concentration of 0.01% H_2_O_2_ in the probes) was added for precisely 10 minutes to all sections. Afterwards the sections were rinsed three times in trisamine buffer (pH 7.6). Finally they were mounted on adhesive glass slides (HistoBond^R^, P. Marienfeld, Lauda-Königshofen, Germany) and covered with coverslips using DPX Mounting medium (Sigma-Aldrich, St. Louis, MO, USA).

### Tissue analysis

Anatomical regions were defined according to *The Rat Brain in Stereotaxic Coordinates* by Paxinos and Watson^75^. Specimens were digitalized at a resolution of 0.01 μm/pixel using a Zeiss Axio Z1 imager and Zen software (Zeiss, Jena, Germany). Tissue samples were blinded and randomized.

For the analysis of the dopamine pathway, equidistant brain slices of eight animals were used per group. Seven striatal sections between bregma 2.5 and −1.0 mm, six sections of the amygdala between bregma −2.0 and −3.2 mm, ten sections of the SNC between bregma −4.8 and −5.8 mm, and eight sections of the VTA between bregma −5.2 and −6.0 mm were analysed per animal.

Digitalized specimens were converted to 8-bit grey value images using IrfanView (Irfan Skiljan, Vienna, Austria).

The striatal region was digitally defined and the mean grey value and the area were measured using a script of Matlab software (MathWorks, Natick, MA, USA). In order to measure the mean grey value of the striatal matrix exclusively, a threshold of 120 was defined, resulting in the subtraction of the striosomes.

The amygdala was divided into basal, lateral and central nuclei and the mean grey value and area were measured using the above mentioned Matlab script (MathWorks, Natick, MA, USA).

Normalized reciprocals of the grey values are displayed in the charts in order to clarify the correlation between measured grey value and fibre density.

Neuronal cell count in the SNC and the VTA was performed using the multi-point tool in ImageJ (National Institute of Health, Methesda, MD, USA).

For the analysis of GAD67-, CB- and PV-positive interneurons, six animals were used per group. Analysis was performed in the forelimb region (S1FL) and the barrel field (S1BF) of the primary somatosensory cortex (S1) using eight equidistant sections per neuronal marker and anatomical region between bregma 1.2 and −0.2 and bregma −0.8 and −3.7, respectively.

A region of interest of 500 μm width and variable length comprising all cortical layers was set perpendicular to the white matter at random positions within the above-mentioned anatomical locations. Neurons were counted per cortex layer using the multi-point-tool in ImageJ (National Institute of Health, Methesda, MD, USA).

The thickness of the cortical layers was measured on consecutive Nissl stained sections perpendicularly to the white matter using ImageJ (National Institute of Health, Methesda, MD, USA).

PV- and GAD67-positive interneurons in the dorsal striatum were counted in three sections per animal from bregma 1.2, 0.6 and 0.0 in a region of interest of 1 mm^2^ and in two sections of the anterior hippocampus (bregma −3.0 and −4.0). The hippocampus was devided in its subregions CA1, CA3 and gyrus dentatus. The cell count was performed as described above.

Volumes of the CC and the anterior hippocampus were estimated using the Cavalieri’s principle on PV-immunostained slides of six animals per group^56^,^57^. Stereological analyses was performed on four sections per animal from bregma 2.0, 0.5, −1.0, and −2.5, in the CC and two sections per animal from bregma 2.7 and 3.6 in the hippocampus. A stereological counting grid was placed over the specimens and volumes were calculated from Cavalieri’s principle:


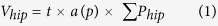



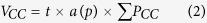


in which V_hip_ and V_CC_ are the volumes of the hippocampus and the CC, t is the slice thickness, a(p) the area associated with each point in the grid and ΣP_hip_ and ΣP_CC_ are the total number of points hitting the hippocampus and the CC, respectively.

### Statistical analysis

All data were tested for normal distribution using D’Agostino-Pearson omnibus normality test and Kolmogorov-Smirnov normality test and two-tailed unpaired t-test was applied. Statistical analysis was performed using GraphPad Prism 6 software (GraphPad, La Jolla, CA, USA).

## Additional Information

**How to cite this article**: Hamburg, H. *et al.* Simultaneous effects on parvalbumin-positive interneuron and dopaminergic system development in a transgenic rat model for sporadic schizophrenia. *Sci. Rep.* **6**, 34946; doi: 10.1038/srep34946 (2016).

## Supplementary Material

Supplementary Information

## Figures and Tables

**Table 1:** Morphological characteristics of DISC1 animal models.

	Morphological Characteristics of DISC1 models								
		Ventricles	Cortex	TH	PV	CB	GAD67	CC	Hipp
mDisc1(∆2–3)	Kuroda *et al.* 2011		=					=	=
	Nakai *et al.* 2014				=				=
	Umeda *et al.* 2016				↓	↓			
mDisc1(129S6/SvEv)	Koike *et al.* 2006		=						=
	Kvajo *et al.* 2008				=				=
	Juan *et al.* 2014	↑	=					=	
mDisc1(∆9–13)	Shen *et al.* 2008	↑	↓		↓ *, ↑ **, = ***, ↓ Hipp			↓	
hDISC1(1–597)	Hikida *et al.* 2007	↑			↓	=			
	Ibi *et al.* 2010				=				
inducible-hDISC1(1–597)	Pletnikov *et al.* 2008	↑							
	Ayhan *et al.* 2010	↑	↓		↓				
	Abazyan *et al.* 2010	↑							
mDisc1-Q31L	Clapcote *et al.* 2007		↓						
	Chandran *et al.* 2014								=
	Borkowska *et al.* 2016						=		
mDisc1-L100P	Clapcote *et al.* 2007		↓						
	Chandran *et al.* 2014								↓ GD
	Borowska *et al.* 2016				↓		=		
tgDISC1 rat	Trossbach *et al.* 2016	↑							
	this manuscript		=	↓	↑*, ↓ **, = ***, ↓ St	↓ ***	=	↓	↑

The table summarizes relevant morphological characteristics of DISC1 animal models.

Abbreviations: *inner layers, **outer layers, ***total, CC = corpus callosum, GD = gyrus dentatus, Hipp = hippocampus, St = striatum.
