# Supplementary material for: Simultaneous effects on parvalbumin-positive interneuron and dopaminergic system development in a transgenic rat model for sporadic schizophrenia
Source: Sci Rep. 2016 Oct 10;6:34946. doi: 10.1038/srep34946 (PMC5056355; doi:10.1038/srep34946)
Supplement: Supplementary Information [file srep34946-s1.doc]

**Supplementary information:**

**Simultaneous effects on parvalbumin-interneuron and dopaminergic system development in a transgenic rat model for sporadic schizophrenia**

Hannah Hamburg, Svenja V. Trossbach, Verian Bader, Caroline Chwiesko, Anja Kipar, Magdalena Sauvage, William R. Crum, Anthony C. Vernon, Hans Jürgen Bidmon, Carsten Korth

**Supplementary Figure 1**

**
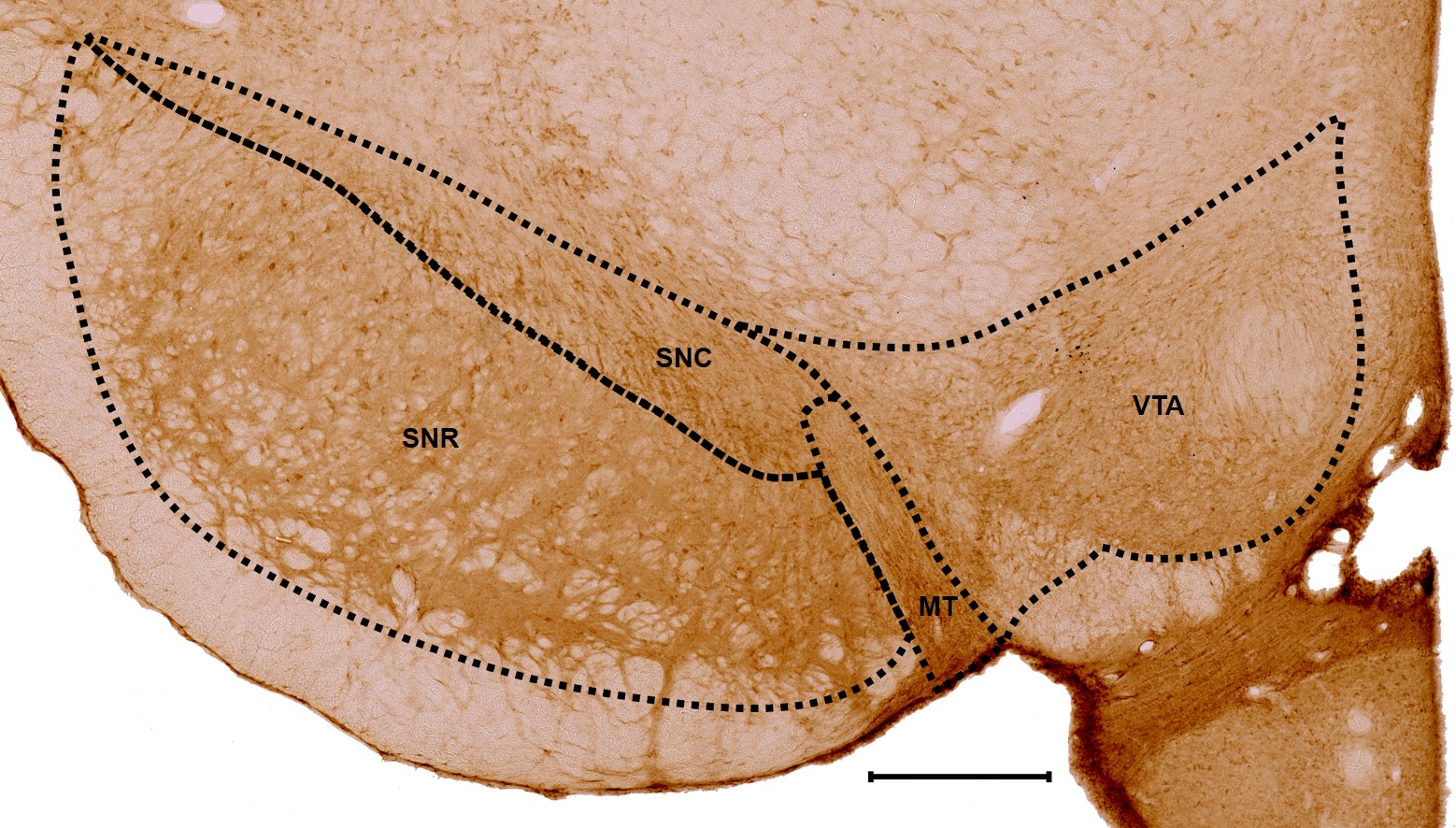
**

**DISC1 expression in the SN and the VTA**

DISC1-staining of a tgDISC1 rat, showing a magnification of the SN and the VTA (for anatomical orientation see also Figure 2). Note the intracellular staining of neurons in both the SN as well as the VTA. Bar 500µm.

Abbreviations: MT = medial terminal nucleus of the accessory optic tract; SNC = substantia nigra compacta; SNR = substantia nigra reticulata; VTA = ventral tegmental area

**Supplementary Figure 2**


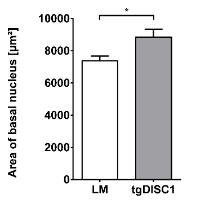

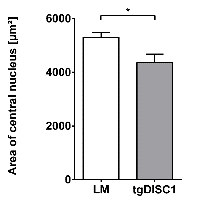

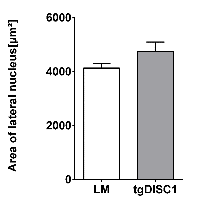


**A**

**B**

**C**

**Surface area of the lateral, basal and central amygdalar nucleus.** (A) Area of the lateral amygdalar nucleus on TH-stainings. No difference is detected between the two groups. (B) Area of the basal amygdalar nucleus. TgDISC1 rats show an increase in surface area of the basal nucleus (*p*= 0.0213). (C) Area of the central amygdalar nucleus. TgDISC1 rats have a decreased surface area of the central nucleus (*p* = 0.0197).

**Supplementary Figure 3**

**
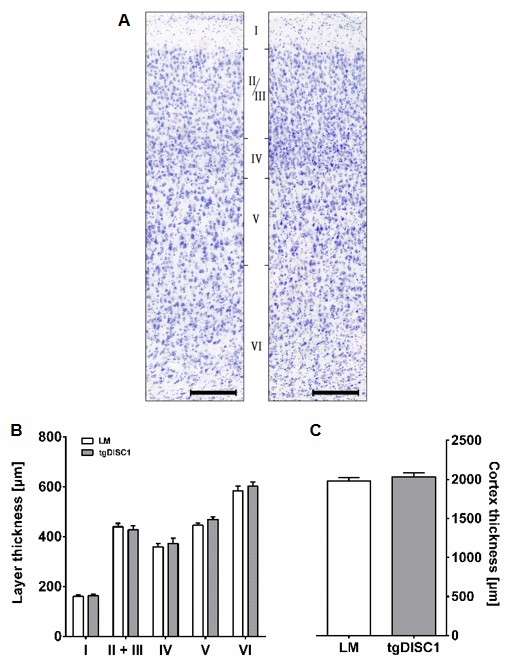
**

**Cortical layer thickness in the SSC.** (A) Magnifications of a Nissl stain of the SSC of a LM (left panel) and a tgDISC1 rat (right panel), bars 250µm. (B) Layer thickness of individual cortex layers and (C) overall cortex thickness. No significant difference was observed between the two groups. Measurements are displayed in µm ± s.e.m.

**Supplementary Figure 4**

**
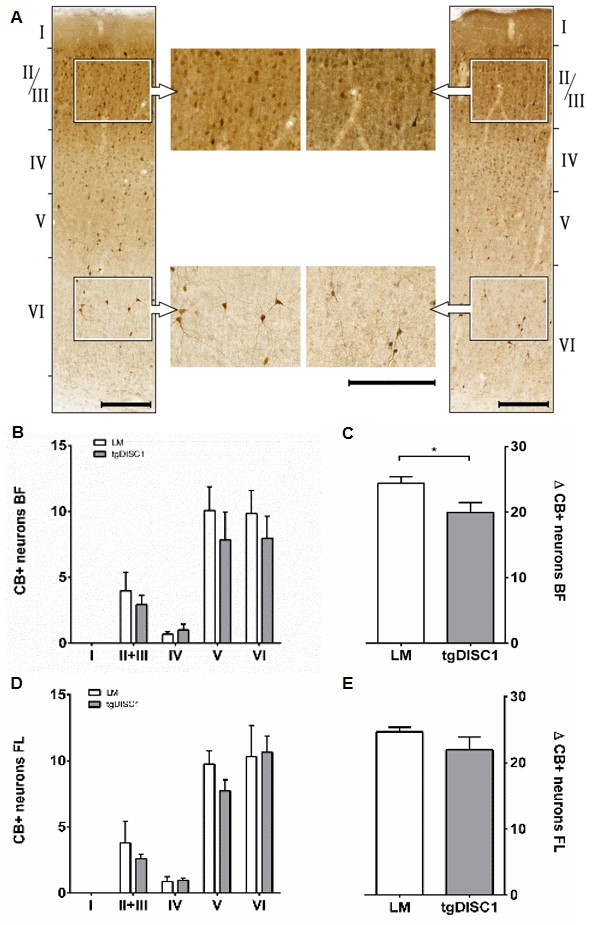
**

**Distribution of Calbindin (CB)-positive interneurons in the SSC.** (A) ROIs from the barrel field of a LM (left panel) and a tgDISC1 rat (right panel) with magnifications in the centre, bars 250µm. (B) CB-positive neurons per cortex layer in the BF. No significant difference in the distribution of CB-positive interneurons is detected between the two groups. (C) Total cell count of CB-positive neurons in the BF. TgDISC1 rats show a slight decrease in total number of CB-positive neurons in the BF (*p* = 0.0338). (D) CB-positive neurons per cortex layer in the FL. No difference in the distribution of CB-positive neurons in the FL is detected between the two groups. (E) Total cell count of CB-positive neurons in the FL. No difference in total number of CB-positive neurons is detected between the two groups.
Cell counts are displayed in number of cells ± s.e.m.

**Supplementary Figure 5**

**
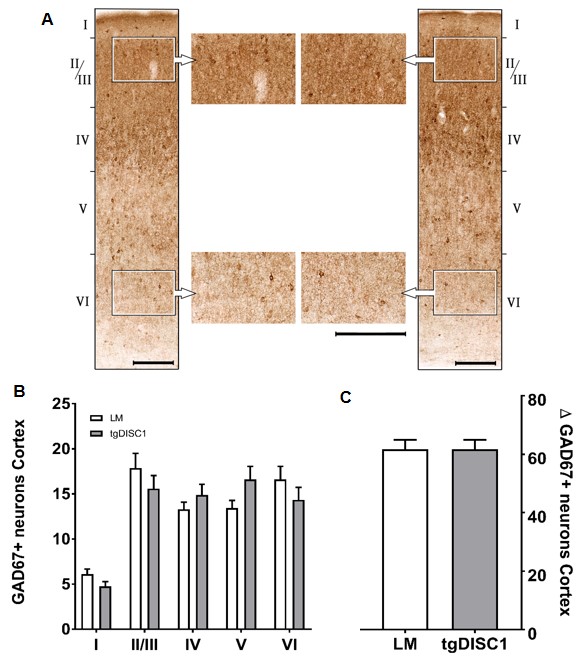
**

**Distribution of GAD67-positive interneurons in the SSC.** (A) ROIs from the barrel field of a LM (left panel) and a tgDISC1 rat (right panel) with magnifications in the centre, bars 250µm. (B) GAD67-positive neurons per cortex layer. No significant difference in the distribution of GAD67-positive interneurons is detected between the two groups. (C) Total cell count of GAD67-positive neurons. No difference in total number of GAD67-positive neurons is detected between the two groups.
Cell counts are displayed in number of cells ± s.e.m.

**Supplementary Figure 6**


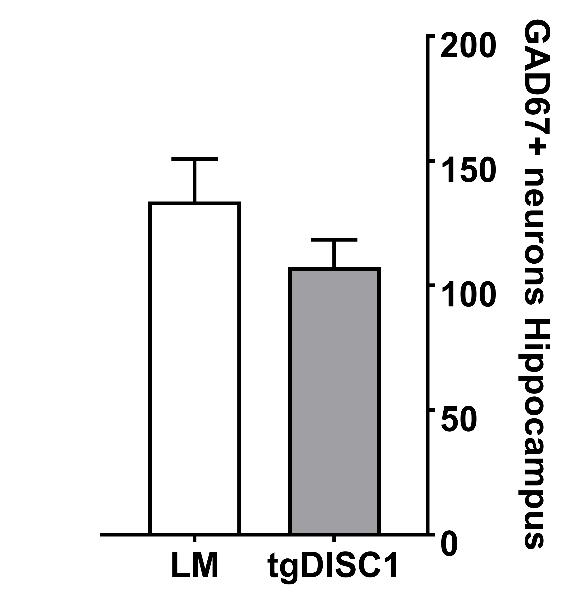

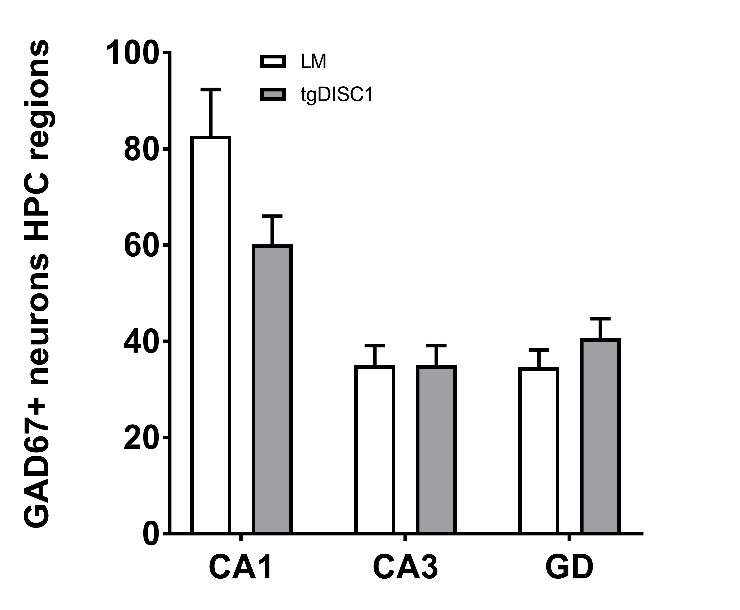

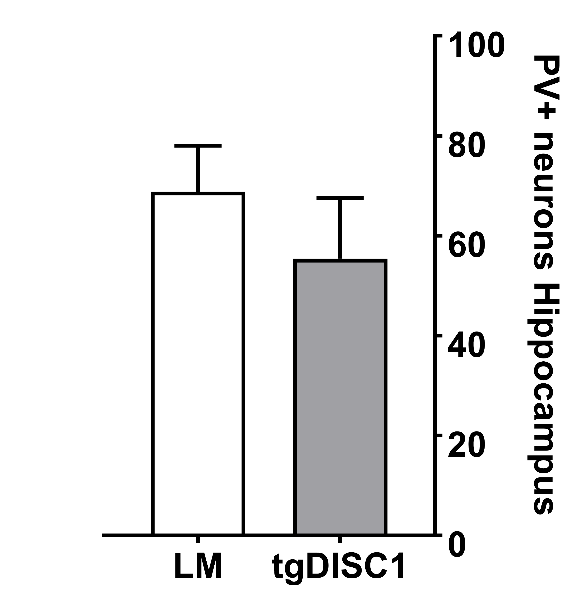

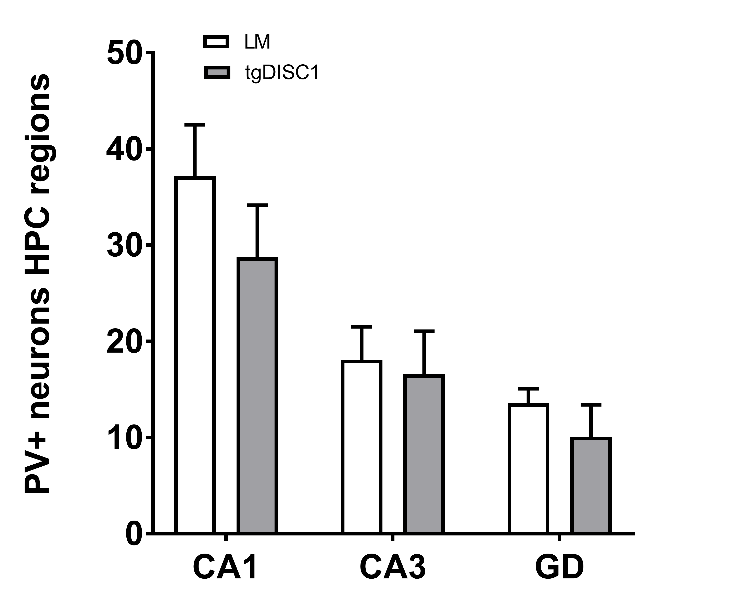


**Distribution of PV- and GAD67-positive interneurons in the Hippocampus.** (A) PV-positive neurons per HPC (Hippocampus) region and (B) total cell count of PV-positive neurons in the Hippocampus. (C) GAD67-positive neurons per HPC region and (D) total cell count of GAD67-positive neurons in the Hippocampus. No significant difference was detected for either marker between the two groups.
